# Characterization of Walnut Oil and Evaluation of Its Neuroprotective Effects in an In Vitro Model of Parkinson’s Disease

**DOI:** 10.3390/molecules29235718

**Published:** 2024-12-04

**Authors:** Monica Filaferro, Rossella Avallone, Cecilia Rustichelli, Giovanni Vitale

**Affiliations:** 1 Department of Biomedical, Metabolic and Neural Sciences, University of Modena and Reggio Emilia, 41125 Modena, Italy; monica.filaferro@unimore.it; 2 Department of Life Sciences, University of Modena and Reggio Emilia, 41125 Modena, Italy; rossella.avallone@unimore.it (R.A.); cecilia.rustichelli@unimore.it (C.R.)

**Keywords:** walnut oil, omega 3 fatty acids, in vitro Parkinson’s models, HMC3 and SH-SY5Y cellular lines

## Abstract

Parkinson’s disease (PD) is a common neurodegenerative disorder marked by the degeneration of dopaminergic neurons and the buildup of α-synuclein aggregates. The current treatments focus on symptom relief, with no drugs available to halt disease progression. This has prompted interest in plant-based extracts as alternative therapies. This study examines the neuroprotective and antioxidant effects of walnut oil (WO), extracted from *Juglans regia* L., in an in vitro PD model using the neurotoxin rotenone (ROT). WO, rich in polyunsaturated fatty acids (PUFAs), including linoleic acid (LA) and α-linolenic acid (ALA), together with minor bioactive components, is known for its neuroprotective properties. Using human HMC3 microglial and SH-SY5Y neuroblastoma cells, we tested WO’s effects on ROT-induced toxicity. The experiments were performed at different time points. The results showed that the co-administration of WO with ROT significantly improved cell viability and reduced reactive oxygen species (ROS) levels. Additionally, conditioned media from WO-treated HMC3 cells enhanced SH-SY5Y cell survival, indicating positive microglia–neuron interactions. Cell viability appeared to be concentration- and time-dependent. These findings highlight WO’s potential, mainly due to its PUFA content, as a promising candidate for preventing neurodegenerative diseases like PD; they underscore the potential of WO content in food for the prevention of neurodegenerative diseases such as PD.

## 1. Introduction

Parkinson’s disease (PD), a synucleinopathy, affects approximately 10 million people worldwide [1,2] and is the second most common neurodegenerative disorder associated with aging. It impacts around 3% of individuals over 65, increasing to 5% by age 85 [3].

PD is marked by the degeneration of dopaminergic neurons in the substantia nigra pars compacta (SNpC) and the formation of Lewy bodies, causing typical motor symptoms. It is a multifactorial disorder influenced by genetic and environmental factors, with age as the primary risk factor and genetics contributing to 5–10% of the cases, often with autosomal inheritance.

Chronic stress and exposure to environmental toxins, such as MPTP, paraquat, and rotenone, which impair mitochondrial function, are also associated with PD [4].

To develop new treatments, researchers rely on animal and cellular models that replicate key aspects of PD, including dopaminergic neuron loss and alpha-synuclein aggregation.

Neurodegenerative diseases like PD are characterized by the progressive loss of neurons in the central nervous system (CNS), a process that is closely linked to neuro-inflammation. In this context, key cells in the CNS—particularly microglia and astrocytes—become activated and release pro-inflammatory cytokines in response to various stimuli. Research has shown that these inflammatory pathways play a central role in the development and progression of neurodegenerative diseases [5].

Microglia, which account for about 5–12% of the cells in the CNS, are especially important in neuro-inflammation. Originating from embryonic myeloid precursors, microglia can exist in three different states: M0 (resting), M1 (pro-inflammatory), and M2 (anti-inflammatory). When microglia become excessively activated in the M1 state, they release pro-inflammatory molecules that exacerbate neuro-inflammation, which in turn accelerate the progression of diseases like PD.

Given the central role of microglia in neuro-inflammation, there is growing interest in modulating their activation and polarization as a potential therapeutic strategy. Shifting microglial polarization from the harmful M1 state to the protective M2 state could help reduce inflammation and slow down the neurodegenerative processes associated with PD and other similar conditions [6,7].

Reactive oxygen species (ROS) are byproducts of mitochondrial activity. While they play important roles in regulating cellular processes such as transcription and cell differentiation, their levels can become problematic when produced in excess. Under the conditions of oxidative stress, this surplus of ROS can lead to significant cellular damage. In PD the situation is even more complicated, as heightened oxidative stress, combined with defects in mitochondrial complex I, results in increased oxidative damage, particularly in the substantia nigra, a brain region that is crucial for movement control [8,9]. Post-mortem studies have shown that oxidative stress is a key contributor to the loss of neurons in PD, a finding that is further supported by the depletion of antioxidants, such as glutathione, in individuals with the disease [10,11].

Current pharmacological treatments for PD primarily focus on managing symptoms, as there are no available therapies that can halt or reverse disease progression. This limitation has led to increasing interest in exploring plant-based extracts as potential therapeutic options.

Among these, natural antioxidant compounds have gained attention for their potential benefits.

Walnut oil (WO) is a highly valued oil extracted from the walnut kernel, widely utilized in both food and healthcare applications. With the increasing focus on health and wellness in recent years, WO has gained popularity among consumers due to its unique health-promoting properties.

The primary appeal of WO lies in its exceptional nutritional profile, particularly its rich composition of fatty acids and beneficial minor components. Its antioxidant capacity is also a key factor in evaluating its nutritional value.

In this regard, WO is of particular interest, as it is well known for being rich in natural antioxidant compounds due to its high percentage of polyunsaturated fatty acids (PUFAs), contributing to approximately 70% of the percentage (*w*/*w*) of the entire oil composition, together with the presence of micro-components such as tocopherols, phytosterols, squalene, and polyphenols which correspond to enhanced antioxidant activity. All the compounds found in WO contribute to significant health benefits in humans [12,13].

To date, over 500 lipid species have been identified in WO, with triacylglycerols and diglycerides being the predominant classes [14]. Fatty acids represent the major constituents of both di- and triglycerides (TAGs), as well as their metabolites, positioning WO as an excellent source of essential fatty acids. In fact, the conversion of TAGs into free fatty acids occurs in both in vivo and in vitro metabolic processes through the action of lipases.

PUFAs are known to help reduce the pro-inflammatory activity induced by lipopolysaccharides in microglia, the immune cells of the brain. When microglia are overly activated or remain activated for extended periods, they can impair neuronal function and cause significant damage to neurons. Omega-3 PUFAs may help protect microglial function and regulate their activity, which is especially important in chronic neurodegenerative diseases like PD. By reducing oxidative stress and helping to balance redox processes, omega-3 fatty acids can lessen the damage caused by reactive oxygen species (ROS) and reactive nitrogen species. These harmful molecules can otherwise oxidize vital cellular components such as DNA, proteins, and lipids, contributing to neurodegeneration in the CNS [15].

Cellular models of PD offer valuable insights into the underlying mechanisms of the disease, including alpha-synuclein misfolding, mitochondrial dysfunction, and disruptions in axonal transport [16]. These models are particularly useful for studying how PD develops at the cellular level, and the administration of neurotoxins can help replicate cellular conditions that mimic the disease.

In our study on PD, we chose the HMC3 (Human Microglia Clone-3) cell line, which was developed through the SV40-dependent immortalization of embryonic microglial cells derived from the primary cultures of 8- to 12-week-old human cortical and spinal cord cells. This cell line is especially valuable because it responds to neurotoxins like rotenone, MPTP, and paraquat, making it an ideal model for investigating microglial activation in PD [17].

Additionally, we included SH-SY5Y cells in our research, which are widely used in PD studies due to their versatility. These cells can grow both in adhesion and suspension and have the ability to differentiate into dopaminergic neurons. This makes SH-SY5Y an excellent choice for studying the neuronal aspects of PD, including neurodegeneration [16,18,19].

Rotenone (ROT) is a lipophilic compound derived from tropical plants that can cross the blood–brain barrier (BBB). It inhibits mitochondrial complex I, leading to a decrease in ATP production and an increase in ROS, both of which mimic the pathology of PD [20,21]. This makes ROT an ideal model for studying neuroprotection in PD.

In addition to neurotoxins, walnuts have garnered attention for their impressive nutritional profile. They are highly caloric and rich in essential nutrients, particularly natural antioxidants, which have been shown to protect against certain types of cancer and reduce the risk of cardiovascular disease. Walnuts are also a significant source of polyunsaturated fatty acids (PUFAs), which have been extensively researched for their health benefits.

Given these properties, the focus of our study was to investigate the potential role of *Juglans regia* (walnut oil), a phytochemical extract that is abundant in PUFAs and other antioxidant components and known for its powerful neuroprotective effects.

Our research aimed to evaluate the neuroprotective and antioxidant effects of WO in an in vitro PD model using the neurotoxin ROT to induce neurodegeneration. We decided to use WO as a whole to get closer to possible WO supplementation conditions in humans. Since the predominant part (up to 99%) of WO consisted of fatty acids, the lipid composition of the WO was characterized through an LC-QO-MS analysis to ensure accurate formulation.

HMC3 microglial cells and SH-SY5Y neuroblastoma cells were used to mimic a simplified model at the CNS level. Moreover, to explore whether microglial cells could provide neuroprotection to neurons, we used conditioned media (CM) from ROT-treated HMC3 cells and applied them to SH-SY5Y cells. This allowed us to assess the impact of microglial secretions on the viability of neuronal cells under oxidative stress.

Cell viability was measured at multiple time points to track the effects over time and to obtain pharmacokinetic data regarding WO for the assessment of better conditions for neuroprotective recovery from damage.

We then evaluated whether WO could mitigate the possible ROS-induced damage and protect cell viability.

These findings open promising new avenues for the use of WO in the treatment of neurodegenerative diseases like PD.

## 2. Results

### 2.1. Identification and Quantification of Fatty Acids in WO

**Figure 1:**
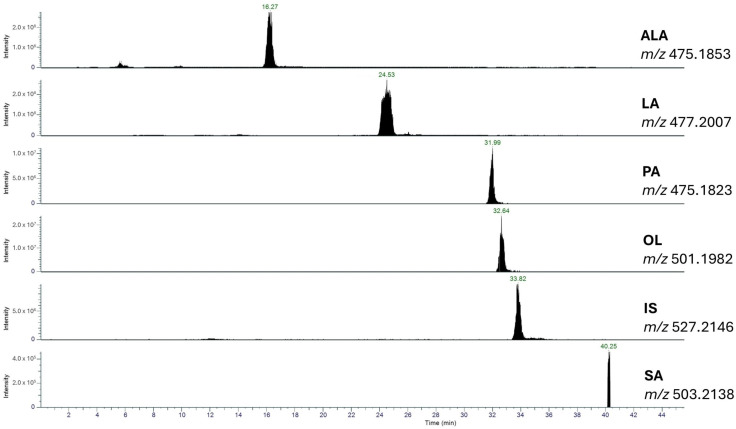
Representative LC-QO-MS chromatogram of a WO sample with the selected quantifier ALA: linolenic acid; LA: linoleic acid; PA: palmitic acid; OL: oleic acid; IS: internal standard; SA: stearic acid.

WO was analyzed using LC-QO-MS (Figure 1) following the saponification and derivatization of its triglyceride fraction, which represents 99% of the total lipid content. The analysis showed that WO is rich in linoleic acid (LA) at a concentration of 591.9 ± 15.87 µg/mg, followed by oleic acid (OL) at 160.7 ± 2.67 µg/mg, and alpha-linolenic acid (ALA) at 106.4 ± 2.98 µg/mg. Saturated fatty acids were present at lower levels, with palmitic acid (PA) at 66.76 ± 1.09 µg/mg and stearic acid (SA) at 26.18 ± 0.95 µg/mg. These values, expressed as the mean ± standard error of the mean (SEM) from three independent experiments, correspond to the following percentage composition: LA at 59.12 ± 1.29%, OL at 16.01 ± 0.12%, ALA at 10.24 ± 0.27%, PA at 6.28 ± 0.10%, and SA at 2.57 ± 0.12%.

The overall fatty acid profile of WO was determined to be 8.9% saturated fatty acids (SFAs), 16.0% monounsaturated fatty acids (MUFAs), and 69.4% polyunsaturated fatty acids (PUFAs), closely matching the reported nominal values of 9.1% SFAs, 16.5% MUFAs, and 69.9% PUFAs. This strong alignment with the nominal composition underscores the reproducibility and reliability of the fatty acid profile of WO and confirms the robustness of the analytical method employed.

Furthermore, this lipid composition—particularly the high PUFA content—highlights WO’s potential nutritional and bioactive properties, as PUFAs like LA and ALA are essential fatty acids known for their health benefits. These findings provide a comprehensive understanding of WO’s lipid profile, supporting its value in both dietary and medicinal applications.

### 2.2. Effect of Rotenone Treatment on HMC3 Cells

**Figure 2:**
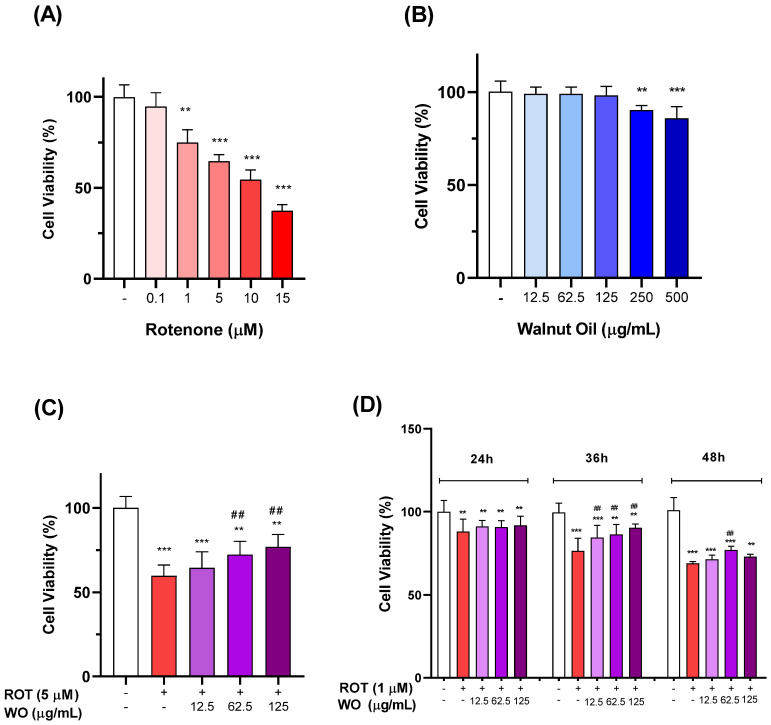
Effects of Rotenone (ROT), WO (WO), and their combination on HMC3 cellular viability. Effects on HMC3 cellular viability by increasing concentrations of ROT (**A**); by increasing concentrations of WO (**B**); by WO on rotenone-induced HMC3 cell toxicity (**C**); by WO on rotenone (1 μM)-induced HMC3 cell toxicity at different times (**D**). All data are expressed as mean ± standard error (from three separate experiments), significance was reported by one-way analysis of variance (ANOVA) followed by Bonferroni multiple comparison test, as compared to control *p* < 0.01 (**), *p* < 0.001 (***); as compared to toxic agent, *p* < 0.01 (##).

The day after cell seeding, the culture medium was replaced as part of the pretreatment protocol. After 24 h, increasing the concentrations of rotenone (0.1, 1.0, 5.0, 10.0, and 15 µM) were administered. The treatment plate was incubated for 48 h, after which cell viability was assessed using the MTT assay. The results indicated that the 48 h treatment (Figure 2A) resulted in a 35% reduction in viability at 5 µM ROT. The 48 h time point was selected based on previous studies examining the time-dependent effects of ROT, with 48 h showing significant outcomes, if compared to 24 h [22].

### 2.3. Effect of Walnut Oil Extract Treatment on HMC3 Cells

The day after cell seeding, the culture medium was removed and replaced with WO at increasing concentrations: 12.5, 62.5, 125, 250, and 500 µg/mL. Following an initial 24 h pretreatment, the WO treatment was continued for an additional 48 h, after which cell viability was assessed using the MTT assay. After 48 h, cells showed a significant reduction in viability at WO concentrations of 250 and 500 µg/mL (Figure 2B). Consequently, ineffective WO concentrations of 12.5, 62.5, and 125 µg/mL were selected for the subsequent experiments.

### 2.4. Impact of Walnut Oil on Rotenone-Induced Cytotoxicity in HMC3 Microglial Cells

A concentration of 5 µM ROT was selected for MTT assays at 48 h, as it caused a marked 35% reduction in cell viability. Cotreatment with WO at concentrations shown to be non-cytotoxic in microglia significantly improved viability, increasing it by 12% at 62.5 µg/mL WO and by 17% at 125 µg/mL WO if compared to ROT alone (Figure 2C). Notably, cotreatment with intermediate and higher concentrations of WO significantly enhanced viability compared to ROT alone.

To further assess WO’s protective effects at a lower ROT concentration, experiments were conducted with ROT at 1 µM and WO cotreatments over a time course of 24, 36, and 48 h. After 24 h, ROT at 1 µM reduced cell viability by 12% relative to controls, with no significant modulation by any WO concentration. After 36 h, ROT at 1 µM reduced viability by 25% relative to control, while cotreatment with WO at 12.5 and 62.5 µg/mL increased viability by approximately 10%, and at 125 µg/mL, viability increased by 18%. After 48 h, ROT at 1 µM reduced viability by 30% compared to controls; cotreatment with WO at 62.5 µg/mL resulted in a slight but significant 7% increase in viability, while the other WO concentrations had no effect.

Figure 2D presents the grouped effects of 1 µM ROT, WO, and their combinations across the three time points, with percentage viability values normalized to each time-matched control. The results indicate that WO’s protective effect is greatest at 36 h and is maximized at the highest WO concentration. These findings guide the selection of optimal conditions—ROT concentration, WO concentration, and treatment duration—for evaluating the neuroprotective effects of WO in this PD model with HMC3 cells. Across all the time points, ROT treatments significantly reduced cell viability.

### 2.5. Evaluation of Reactive Oxygen Species Production in HMC3 Cells Treated with Rotenone and/or Walnut Oil

The evaluation of ROS in the HMC3 cells was conducted using the fluorochrome H2-DCFDA. The cells were pretreated for 6 h with either WO at 125 µg/mL or with culture medium only, followed by a 15 h treatment with either 5 µM ROT or ROT combined with WO. At the end of the treatments, the cells were incubated with H2-DCFDA at a concentration of 10 µM for 40 min. After incubation, excess fluorochrome was removed.

**Figure 3:**
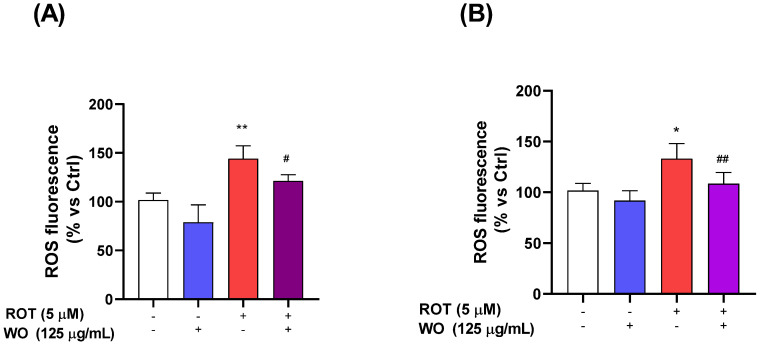
Effects of ROT, WO, and their combination on reactive oxygen species (ROS) levels in HMC3 cells (**A**) and in SHSY5Y cells (**B**). All data are expressed as mean ± standard error (from three separate experiments), significance was reported by one-way analysis of variance (ANOVA) followed by Bonferroni multiple comparison test, as compared to control *p* < 0.05 (*), and *p* < 0.01 (**); as compared to toxic agent, *p* < 0.05 (#), *p* < 0.01 (##).

Plate readings revealed that treatment with WO did not significantly alter ROS levels compared to controls, while ROT increased ROS levels by 45%. Notably, the combination of ROT and WO effectively reduced ROT-induced ROS levels, restoring them to control values (Figure 3A).

### 2.6. Effect of Rotenone Treatment on SH-SY5Y Cells

The experiments involved seeding the SH-SY5Y cells in 96-well plates at a density of 25,000 cells per well, followed by incubation at 37 °C for 24 h. The following day, the culture medium was removed and replaced to simulate pretreatment for an additional 24 h, which is necessary for the subsequent experiments. ROT was then added to the cells to achieve final concentrations of 0.1, 0.5, 1, 5, 10, and 20 µM.

**Figure 4:**
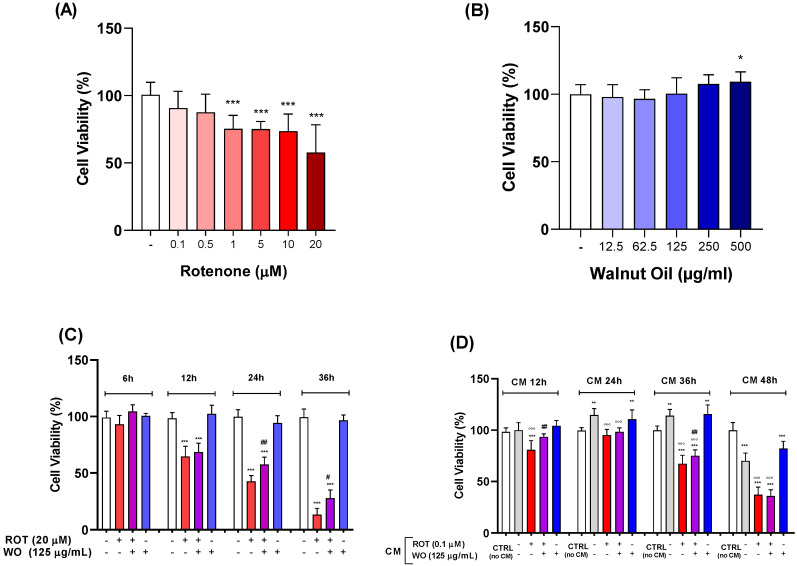
Effects of ROT, WO, and their combination on cellular viability in SH-SY5Y cells. Effects on cellular viability by increasing concentrations of ROT (**A**); of WO (**B**); by WO after ROT-induced cell toxicity (**C**); effects on cell viability after non-contact co-culture with CM from HMC3 treated with rotenone and/or WO (**D**). Data are means ± standard error (*n* = 3) ANOVA followed by Bonferroni test, as compared to control *p* < 0.05 (*), *p* < 0.01 (**) and *p* < 0.001 (***); as compared to toxic agent, *p* < 0.05 (#) and *p* < 0.01 (##); as compared to CM control, *p* < 0.001 (°°°).

The results from the MTT assay indicated that treatment with ROT for 48 h (Figure 4A) led to significant decreases in cell viability, with a 25% reduction observed at a concentration of 1 µM. At the highest concentration of 20 µM, cell viability decreased by 43%. Based on these findings, ROT at 20 µM was selected for evaluating the protective effect of WO, as it markedly reduced cell viability.

### 2.7. Effect of Walnut Oil Extract Treatment on SH-SY5Y Cells

The cells were pretreated for 24 h with WO at concentrations of 12.5, 62.5, 125, 250, and 500 μg/mL. Similarly to the HMC3 cells, this pretreatment is essential for evaluating the protective effect of WO against ROT. After the pretreatment, WO was added to the cells at the aforementioned concentrations to simulate combination treatments and incubation followed for an additional 24 h, after which cell viability was assessed using the MTT assay. The results, shown in Figure 4B, indicate that only the 500 μg/mL concentration of WO significantly altered cell viability, increasing it by approximately 9% when compared to the control.

### 2.8. Combined Effects of Rotenone and Walnut Oil Treatment on SH-SY5Y Cells

To evaluate the potential neuroprotective effect of WO against rotenone-induced toxicity, the SH-SY5Y cells were treated with 20 µM rotenone, a concentration known to significantly decrease cell viability, in combination with 125 μg/mL of WO. This experiment involved a 24 h pretreatment with WO, followed by the administration of ROT and/or WO at various time points (6, 12, 24, and 36 h) to assess the impact of time on the potential protective effects of WO against ROT.

The results from the 6 h treatment indicated no significant effects compared to controls. At the 12 h mark, both the ROT and ROT + WO treatments similarly reduced cell viability by 30–35%, indicating that the combination did not provide any recovery in viability. After 24 h of treatment, ROT significantly decreased cell viability to 43% of the control levels, while the ROT + WO combination restored viability to 57%, demonstrating a statistically significant difference. At 36 h, ROT drastically reduced cell viability to just 13% of the control levels; however, the addition of WO significantly increased viability to 27%.

Figure 4C summarizes the whole data across the various treatment durations, highlighting that the effect of rotenone is time-dependent and that the protective effect of WO becomes evident at the 24 h mark.

### 2.9. Evaluation of Reactive Oxygen Species Production on SHSY5Y Cells Treated with Rotenone and/or WO

To further characterize the protective mechanisms, the ability of WO to counteract rotenone (ROT)-induced intracellular reactive oxygen species (ROS) production in the SH-SY5Y cells was investigated using the H2-DCFDA assay following a 15 h exposure. As illustrated in Figure 3B, treatment with WO at a concentration of 125 µg/mL did not reduce intracellular ROS levels, whereas ROT at 5 µM significantly increased ROS levels by 33%. However, the combination of WO and ROT effectively restored ROS production to control levels.

### 2.10. Impact of Conditioned Medium from HMC3 Cells on SH-SY5Y Cell Viability

This experiment aimed to evaluate potential “cross-talk” between two human cell lines: HMC3 microglial cells and SH-SY5Y neuroblastoma cells. The evaluation involved simulating a co-culture between the cell lines to determine whether substances released into the treatment medium from the HMC3 cells could influence the viability of the SH-SY5Y cells.

In this experiment, conditioned media obtained from the HMC3 cells treated for 12, 24, and 48 h were added to the SH-SY5Y cells for a duration of 24 h. The plate seeded with the HMC3 cells was pretreated with WO at a concentration of 125 µg/mL, followed by treatment with either WO or ROT combined with WO, as well as a control medium for the subsequent ROT treatments.

Six hours after pretreatment, the HMC3 cells were treated with 0.1 µM ROT and/or WO 125 µg/mL. The treatments of HMC3 with ROT and/or WO were conducted for 12, 24, 36, and 48 h. After these treatments, the conditioned medium from the HMC3 cells was removed and used to treat the SH-SY5Y cells for an additional 24 h, after which cell viability was assessed using the MTT assay.

The used concentration of ROT was not effective in changing either the HMC3 or SH-SY5Y cell viability and was selected on the basis of our previous experiments [23] as well as other studies [24,25] in order to stimulate microglia activation cells and to investigate the cross-talk between the two cell lines occurring with regard to cell viability. Although the cell viability was not affected by ROT in the HMC3 cells, the CM ROT from microglia promoted damage to neuroblastoma cells.

Figure 4D presents the results grouped by the four treatment durations of the HMC3 cells, expressed as percentages of viability for SH-SY5Y compared to controls. Statistical analysis indicated that the metabolic activity of the conditioned medium control (CM CTRL) obtained from the untreated HMC3 cells at 12 h did not differ significantly from the control treatments (no CM). In contrast, the CM from the HMC3 cells treated with 0.1 µM ROT exhibited a 20% reduction in viability compared to both CTRL and CM CTRL. However, at this time point, the CM from the ROT + WO-treated HMC3 cells demonstrated increased viability compared to the CM from the ROT-treated cells, with no significant difference observed from either CTRL or CM CTRL. This suggests that the adverse effects of ROT were completely reversed by the combination with WO.

At 24 h, treatment with CM significantly increased the metabolic activity of the SH-SY5Y cells compared to the control (CTRL). A similar trend is observed for CM WO, indicating that the supplementation of the HMC3 cells with WO did not alter the viability of the SH-SY5Y cells compared to the values of CM CTRL alone. The results for CM ROT and CM ROT + WO treatments were not significantly different from each other, and both exhibit reduced viability compared to CM CTRL.

The analysis of the treatments collected at 36 h revealed an increase in cell viability following treatment with CM and CM WO when compared to CTRL. Conversely, CM ROT 0.1 showed a significant decrease in viability relative to both CTRL and CM CTRL. Notably, the CM ROT + WO treatment demonstrated a significant increase in viability (8%) compared to CM ROT 0.1.

The results from the conditioned media collected at 48 h indicated that the SH-SY5Y cells treated with CM and CM WO experienced a reduction in metabolic activity of 30% and 20%, respectively, compared to CTRL. The CM ROT treatment induced a drastic reduction in viability compared to both CTRL and CM CTRL, and the ROT + WO combination did not alter the effects of ROT alone, maintaining similar statistical significance.

The evaluation of the experiment across the four time points highlights that at 24 and 36 h, both CM and CM WO significantly enhanced viability compared to CTRL. This increase in viability has also been observed in experiments using 24 h CM from HMC3 cells, suggesting that microglial cells produced factors that promote the vitality and metabolic activity of SH-SY5Y cells at these time points. However, the treatments obtained at 12 h did not elicit this effect. Conversely, both CM and CM WO at 48 h reduced viability compared to the control, suggesting that HMC3 cells may produce substances that negatively impact the viability of the neuroblastoma cell line. It is worth noting that at all the time points, CM and CM WO showed no significant differences from each other, indicating that WO alone does not influence the effects of CM.

The viability of CM ROT treatments was consistently reduced across all the time points compared to CM CTRL, although not compared to CTRL. Specifically, the results from CM ROT at 24 h were not significantly different from CTRL, suggesting that the increase in viability due to CM, at this time, balanced the toxicity of CM ROT. This balance did not occur with the conditioned media from later time points, where viability decreases markedly in a dose-dependent manner.

At 12 h, the combination of CM ROT + WO facilitated a recovery of viability compared to CM ROT; however, this recovery cannot be attributed to the effect of the conditioned medium contrary to previous observations. At 24 h, the values of CM ROT and CM ROT + WO were not significantly different from each other and, as previously noted, were similar to CTRL but lower than CM CTRL. The recovery was, again, evident with the 36 h media, along with a notable reduction in viability associated with CM ROT. Finally, CM ROT and CM ROT + WO collected at 48 h showed no significant differences between them, with both reducing viability to approximately 37%.

## 3. Discussion

Currently, there are no pharmacological therapies available to halt the progression of PD. By the time of diagnosis, a significant loss of dopaminergic neurons has occurred, often affecting multiple regions of the CNS. Given this context, plant-based compounds like WO are being explored for their potential neuroprotective effects as novel therapeutic strategies for preventing and treating PD. This research utilizes both in vivo and in vitro models to assess the efficacy of these compounds in mitigating disease progression.

WO was selected for its high PUFA content, constituting about 70% of its total fatty acids. Clinical and experimental studies have shown promising effects of PUFAs in systemic diseases, including diabetes, cardiovascular disorders, and various neurological conditions [15]. WO’s lipid profile is rich in omega-3 and omega-6 fatty acids, exhibiting significant antioxidant properties, which are evidenced by increased glutathione reductase activity and reduced oxidized proteins, DNA damage, and ROS in animal models fed an omega-3 PUFA-enriched diet. PUFAs may alleviate oxidative stress and inflammation in the CNS, suggesting their potential as a therapeutic strategy for neurodegenerative diseases [26].

It is well documented also in vitro [27,28] that PUFAs, though incorporated mainly into triglycerides, can undergo breakdown to free fatty acids due to the presence of different types of lipase enzymes which hydrolyze the ester at the cellular level so that they can exert their intracellular effects. These enzymes are essential also in the context of the central nervous system (CNS), playing critical roles in both energy metabolism and brain myelination. Although the etiology of PD remains uncertain, excessive ROS production or deficiencies in antioxidant defenses in the brain can induce oxidative stress, leading to neuronal damage and degeneration [29]. In vivo and in vitro models replicating the key pathological features of PD, particularly the degeneration of dopaminergic neurons, have been developed for research [20]. Common experimental models employ neurotoxins, such as rotenone, which selectively induce degeneration in the CNS dopaminergic system, closely mimicking disease pathology.

Omega-3 PUFAs have demonstrated potential in mitigating PD by inhibiting pro-inflammatory cytokine release, restoring mitochondrial function, and reducing endoplasmic reticulum stress. Notably, immunoreactivity for glial cell line-derived neurotrophic factor (GDNF) was elevated in dopaminergic neurons treated with DHA in an MPTP model compared to those treated with MPTP alone [30]. At the cellular level, eicosapentaenoic acid (EPA) was shown to enhance brain-derived neurotrophic factor (BDNF) expression in SH-SY5Y cells exposed to MPP+ [31]. These findings suggest that PUFA supplementation may prevent PD onset or slow its progression through anti-apoptotic mechanisms [32].

ROT, a commonly used neurotoxin for PD modeling, allows the observation of key PD features, including motor deficits, catecholamine depletion, and α-synuclein inclusions, making it valuable for neuroprotection studies.

In previous in vivo studies using Drosophila melanogaster, we examined the neuroprotective effects of WO, chosen for its high PUFA content. Flies were placed on a WO-enriched diet, subjected to ROT treatment alone, or combined with WO. The WO-supplemented flies exhibited improved locomotor activity and reduced mortality compared to those on a regular diet during ROT exposure. Liquid chromatography–mass spectrometry (LC-MS) analysis revealed significant increases in alpha-linolenic acid and linoleic acid levels in flies fed the WO-enriched diet. Additionally, WO-fed flies displayed elevated dopamine (DA) levels, while ROT treatment significantly reduced DA levels. The combined WO + ROT treatment restored DA levels to control values.

In in vitro models with CNS-derived cell lines, rotenone’s primary toxicity mechanism involved inhibiting mitochondrial complex I, leading to disrupted respiration, increased ROS production, and cell death [20,21,33,34,35]. In this study, we used the SH-SY5Y neuronal and HMC3 microglial cell lines, treating them with ROT to assess cytotoxic effects and validate the model. Additionally, WO was administered to determine its potential to enhance cell viability, both alone and in combination with ROT, to explore its protective effects against oxidative damage. We also treated SH-SY5Y cells with conditioned media from HMC3 cells exposed to ROT and/or WO to investigate the microglial influence on neuronal survival in a non-contact co-culture environment.

Following treatment with ROT, we assessed the effect of WO on cell viability across increasing concentrations. After 48 h, the HMC3 cells exhibited a slight yet significant reduction in viability at 250 and 500 µg/mL WO concentrations. In the SH-SY5Y cells, the 48 h treatment with 500 µg/mL WO increased cell viability by 10%.

The HMC3 cells were treated with ROT, at concentrations that significantly reduced viability, either alone or with WO concentrations that did not independently impact viability. Viability experiments included a 24 h pretreatment with increasing WO concentrations or medium alone, followed by exposure to either ROT alone or the ROT + WO combination for an additional 48 h. We then lowered the ROT concentration to 1 µM and co-administered various WO combinations, assessing viability at 24, 36, and 48 h to evaluate the PD model in a dose- and time-dependent manner. The results indicated that WO-mediated recovery began at 36 h and was greater with respect to 48 h.

Corsi et al. [15] investigated the effects of a PUFA-rich supplement on murine BV2 microglial cells cotreated with lipopolysaccharide (LPS). Their findings showed a significant, dose-dependent increase in cell viability compared to LPS-only treatment, restoring viability to control levels. These results support the hypothesis that nutraceutical supplementation with PUFAs, particularly omega-3, may exert protective and regulatory effects on microglial function, aligning with our findings.

To explore the role of oxidative stress in the observed viability changes, we conducted plate fluorometric assays to measure ROS production in both HMC3 and SH-SY5Y cells under conditions similar to the viability experiments, using 5 µM ROT and 125 µg/mL WO alone or in combination, following a 6 h WO pretreatment or medium replacement. The results showed that the WO treatments did not alter the ROS levels compared to controls, while ROT significantly increased the ROS levels. Importantly, the combined ROT + WO treatment effectively mitigated ROT-induced ROS elevation.

Supporting these findings, Cardoso et al. [36] demonstrated that omega-3 deficiency reduces neuronal homeostasis under oxidative stress in the rat nigrostriatal system. They observed increased nitric oxide (NO) and lipid peroxide levels in the striatum, alongside decreased catalase and superoxide dismutase activity in the substantia nigra and striatum. While these results were obtained under different conditions, they suggest that higher omega-3 PUFA intake may confer neuroprotection against oxidative damage [32].

In the SH-SY5Y cells treated with 20 µM ROT, a concentration known to induce significant cell death, combined with 125 µg/mL WO, we observed a significant recovery of cell viability at 24 and 36 h, indicating a protective effect against ROT-induced toxicity.

Substances causing cytotoxicity or apoptosis can affect pharmacokinetics by altering cell behavior. Cell viability tests, like MTT (measuring mitochondrial function), help assess how drugs interact with cells, affecting drug entry, distribution, and elimination. SHSY5Y cells are a useful model for studying CNS drug pharmacokinetics. These assays help determine if a drug harms cells and at what concentrations, providing insights into its safety and pharmacokinetics.

As shown in Figure 4C, if treated with 125 µg/mL WO, the SH-SY5Y cells did not show any significant difference in viability at the time points ranging from 6 to 36 h. This evidence indicates a lack of risk profile for the studied concentration of WO together with information for safety data and cellular effects.

Other studies have investigated the neuroprotective effects of PUFAs on SH-SY5Y cells, such as Alarcon-Gil et al. [37], who pretreated SH-SY5Y cells with linoleic acid and found neuroprotective and anti-inflammatory effects following exposure to 6-OHDA.

In summary, we established a contact-free co-culture model where SH-SY5Y cells were treated with conditioned media from HMC3 cells exposed to ROT, WO, or both. This conditioned media contained factors released by microglial cells, enabling us to assess their effects on neuronal viability. Our results indicate that while the treatment of HMC3 cells with WO alone did not alter the effects of WO-conditioned media on SH-SY5Y cells, the combination of WO with ROT produced a conditioned medium that significantly restored cell viability relative to CM ROT at 36 h. However, CM ROT did not reduce SH-SY5Y cell viability at 24 h, suggesting a potential protective effect from the conditioned medium at this time point.

At 48 h, the conditioned media showed decreased viability across all the treatments, with no recovery in the combined ROT + WO treatment, indicating that this time point may not be optimal for studying HMC3 cell effects on SH-SY5Y cells. Conversely, 12 h conditioned media appeared unaffected by factors from untreated or WO-treated microglial cells, while ROT-conditioned media reduced viability, which fully recovered with the ROT + WO combination.

These findings support the hypothesis that HMC3 cells, in the absence of prior toxic exposure, may secrete factors that enhance neuroblastoma cell viability. Our results align with previous studies examining the impact of BV-2 cell conditioned media on SH-SY5Y cells for other protective compound classes [23,24,38]. Although specific microglial products were not identified in this study, others have suggested that neurotoxic factors, such as superoxide, myeloperoxidase, prostaglandins, NO, glutamate, IL-1b, and TNFa, may play a role in causing toxicity to neuron-like cells under in vitro models [39,40].

Our findings also indicate that in this in vitro model, supplementation with PUFAs from WO exhibits a neuroprotective effect against ROT-induced neurotoxicity. The antioxidant and neuroprotective properties of WO, which is rich in PUFAs, were evaluated, demonstrating efficacy at the cellular level in reducing cytotoxicity and intracellular ROS production in both HMC3 and SH-SY5Y cell lines following exposure to the neurotoxin ROT.

WO is composed of over 90% fatty acids, particularly polyunsaturated fatty acids (PUFAs), which are likely to play a significant functional role. Accordingly, its chemical characterization has primarily focused on its fatty acid profile. However, the unsaponifiable fraction, comprising less than 1% of the oil, contains a diverse array of bioactive minor constituents, including tocopherols, phytosterols, and phenolic compounds. Despite their low concentrations, these components may exert significant biological effects and interact synergistically with the PUFAs, underscoring the importance of investigating their potential contributions to the oil’s overall properties.

## 4. Materials and Methods

### 4.1. Chemicals and Material for Biological Assays

Human HMC3 microglial cells were obtained from ATCC (Manassas, VA, USA), while human neuroblastoma SH-SY5Y cells were sourced from the European Collection of Authenticated Cell Cultures (ECACC). Eagle’s Minimum Essential Medium (EMEM) was also purchased from ATCC (Manassas, VA, USA). Dulbecco’s Modified Eagle Medium—F-12 Nutrient Mixture (DMEM-F12, 1:1) and Hank’s Balanced Salt Solution (HBSS) were provided by Gibco (Thermo Fisher Scientific, Waltham, MA, USA). RPMI 1640 media, with and without phenol red, were obtained from Corning (Corning, NY, USA). Additional reagents including non-essential amino acids, L-glutamine, fetal bovine serum (FBS), penicillin/streptomycin solution, rotenone, DMSO, thiazolyl blue formazan (MTT), sodium dodecyl sulfate (SDS), phosphate-buffered saline (PBS), and H2DCFDA (2,7-dichlorodihydrofluorescein diacetate) were sourced from Invitrogen (Thermo Fisher Scientific, Waltham, MA, USA).

### 4.2. Walnut Oil Preparation and Analysis

WO was extracted via cold pressing from *Juglans regia* L. walnuts cultivated under certified organic farming practices (2022 harvest; CiboCrudo, Milan, Italy), with a reported fatty acid composition of 9.1% saturated fatty acids (SFAs), 16.5% monounsaturated fatty acids (MUFAs), and 69.9% PUFAs. The oil was unrefined, free from high-temperature processing, and tested to confirm the absence of heavy metals, aflatoxins, and molds.

### 4.3. Chemicals and Solvents for Walnut Oil Preparation and Analysis

Fatty acid standards (PA, SA, OL, LA, ALA, eicosadienoic acid as internal standard—IS) were purchased from Merck (Milan, Italy) and stored at −20 °C. The reagents included pBPB, 18-Crown-6, NaOH, KOH, HCl, hexane, MeOH, PPT solution, and HPLC-grade water, acetonitrile, and other solvents, filtered and degassed before HPLC analysis.

### 4.4. Lipid Saponification

Lipid saponification was carried out according to our previous paper [41]. Briefly, 10 mg of WO was combined with IS solution, methanol, and NaOH, then stirred at 80 °C for 1.5 h. After cooling, hexane was added to separate the unsaponifiable fraction. The aqueous phase was acidified with HCl, and free fatty acids were extracted with hexane, evaporated, and dissolved in methanol.

### 4.5. Fatty Acid Derivatization

Free fatty acids were derivatized with p-bromophenacyl bromide (pBPB) for better LC-ESI-MS detection. The samples were mixed with phenolphthalein solution and methanolic KOH, dried, then treated with pBPB and 18-crown-6 in ACN, reacted at 80 °C, centrifuged, and stored at −20 °C [41].

### 4.6. Walnut Oil Analysis (LC-QO-MS)

The WO analysis used an Ultimate 3000 UHPLC and a Q Exactive™ Hybrid Quadrupole-Orbitrap™ mass spectrometer (Thermo Fisher Scientific, Waltham, MA, USA), operating in ESI positive mode with precise settings and calibration [41].

### 4.7. Calibration Curves

Target analyte working solutions (range: 3.0–450 μg/mL for ALA, 15.0–2250 μg/mL for LA, and 13.0–1950 μg/mL for PA, OL, and SA) were added with 20 μL of IS solution (2.5 mg/mL) and analyzed in triplicate. Analyte identification relied on retention times and mass accuracy, with calibration curves created using weighted regression. The R^2^ values were above 0.991, and LLOQ values were below the lowest calibrators.

### 4.8. Cell Cultures

Human HMC3 microglia cells were cultured in EMEM supplemented with 2 mM L-glutamine, 100 U/mL penicillin, 100 µg/mL streptomycin, and 10% FBS. Human neuroblastoma SH-SY5Y cells were maintained in a 1:1 mixture of DMEM and F12 medium, supplemented with 2 mM L-glutamine, 100 U/mL penicillin, 100 µg/mL streptomycin, 1% non-essential amino acids, and 10% FBS.

Both cell lines (ATCC, Manassas, VA, USA) were incubated at 37 °C in a humidified atmosphere with 5% CO_2_. The cell cultures were kept at 80–90% confluence for HMC3 and 70% for SH-SY5Y and were limited to a maximum of 20 passages.

### 4.9. Cell Viability Assay

The HMC3 and SH-SY5Y cells were seeded in 96-well plates at densities of 7500 and 25,000 cells per well, respectively. Cytotoxicity was induced in both cell lines using ROT. WO (WO) was tested at concentrations ranging from 12.5 to 500 µg/mL to assess its effects on cell viability. To investigate the potential protective effects of WO against ROT-induced cytotoxicity, the cells were pretreated with either WO (12.5–125 µg/mL for HMC3 cells and 125 µg/mL for SH-SY5Y cells) or a control medium containing 5% FBS for 24 h. The cells were then treated with either WO, ROT, ROT + WO, or the control medium for various time intervals. ROT was applied at concentrations of 1 and 5 µM for HMC3 cells and 20 µM for SH-SY5Y cells.

Cell viability was determined using the MTT assay, where formazan crystals formed by metabolically active cells were solubilized in SDS, and absorbance was measured at 570 nm with a reference wavelength of 620 nm.

### 4.10. Determination of Reactive Oxygen Species (ROS) in Cells

The HMC3 and SH-SY5Y cells were seeded in 96-well plates at densities of 10,000 and 50,000 cells per well, respectively, and allowed to adhere overnight. The cells were then pretreated with either WO or control medium for 6 h before being treated with ROT, WO, ROT + WO, or control medium for 15 h. ROT was applied at 5 µM in both cell lines, while WO was used at 125 µg/mL. Following treatment, the cells were washed with HBSS and incubated for 45 min at 37 °C with 10 µM H2-DCFDA dye to detect ROS.

After dye incubation, the cells were washed and incubated in HBSS. The ROS levels were quantified using a plate reader (GloMax Discover, Promega, WI, USA) with excitation and emission wavelengths set to 485/520 nm.

### 4.11. Non-Contact Co-Culture Assay with WO and SH-SY5Y Cells

The SH-SY5Y cells were seeded in 96-well plates and adhered for 24 h. The HMC3 cells, plated in 24-well plates, were treated with ROT, WO, ROT + WO, or a control medium for 12, 24, 36, and 48 h. Media from each treatment were centrifuged, and HMC3 supernatants were used to treat the SH-SY5Y cells for 24 h. The SH-SY5Y cell viability was measured with the MTT assay.

### 4.12. Statistical Analysis

Data analysis and graphics were performed using GraphPad Prism v. 10.2.3. The experiments were repeated at least three times, with values shown as mean ± SEM. Group differences were analyzed using one-way ANOVA with Bonferroni post hoc test, considering *p* < 0.05 as statistically significant. References were managed with Zotero.

## 5. Conclusions

Our results confirm the concentration-dependent toxicity of rotenone, which induces cell damage in both cell lines. WO alone did not affect cell viability at the chosen doses; however, when co-administered with rotenone, WO enhanced cell viability in both microglial and neuronal cells. Fluorimetric analyses revealed that rotenone increases ROS production in HMC3 and SH-SY5Y cells, while co-administration with WO significantly reduced ROS levels, especially at higher doses.

Our findings provide insight into some of the mechanisms underlying rotenone toxicity in different cell types, including neuronal progenitors and microglia. Additionally, experiments with “conditioned medium” showed that CM from untreated HMC3 cells slightly increased SH-SY5Y cell viability at 24 and 36 h compared to no CM. Conversely, CM from rotenone-treated cells reduced SH-SY5Y viability from 36 h onward, although this reduction was mitigated when rotenone was combined with WO at 12- and 36 h exposure times. These results suggest an important interaction between microglial and neuronal cells.

Evidence on the neuroprotective action of the lipid component of WO comes from our previous research on a PD model on Drosophila [41] in which it was highlighted that a WO supplementation in the diet was able to change the profile of lipids and, in particular, of PUFAs in the heads of flies. In parallel, flies fed with a diet supplemented with WO showed an improvement in behavioral and biochemical parameters when exposed to rotenone.

Moreover, the choice to administer WO in both in vivo and in vitro studies, rather than pure fatty acids, underscores its potential for translational applications in humans [42,43]. However, to fully elucidate the mechanisms underlying WO’s neuroprotective action, future experiments should focus on the specific contributions of its omega-3 fatty acids, such as alpha-linolenic acid (ALA), in PD models. Furthermore, research on the role of tocopherols and other minor bioactive compounds present in WO is necessary to clarify their relative contributions to its overall effects. Such studies will be crucial for distinguishing the specific roles of fatty acids in neuroprotection. Additionally, detailed analyses of the fatty acid composition in HMC3 and SH-SY5Y cells, both before and after WO treatment, are required to better understand how these lipids influence the observed cellular effects. These approaches will provide critical insights into the molecular interactions driving WO’s therapeutic potential.

There are limitations to our study. Our focus was addressed mainly on fatty acids’ contribution to WO’s neuroprotective role. Our research design was supported by in vivo experiments ascribing the results to changes in PUFAs’ profile at the animal brain level. Therefore, we postulate a significant role of PUFAs, which, as previously mentioned, have demonstrated beneficial effects on behavioral and biochemical parameters.

Further clarification of the processes of microglial activation, their role in inflammation, and WO’s potential influence on these processes is needed. A deeper understanding of how PUFA-rich vegetable oils affect neuron–microglia communication and contribute to CNS homeostasis and integrity could have significant implications for their therapeutic potential in neurodegenerative diseases such as PD.

Additionally, these findings may enhance our understanding of the biochemical and molecular mechanisms contributing to the onset of such diseases.

## Data Availability

The data presented in this study are available upon request from the corresponding authors.
